# Preclinical evaluation of vascular closure devices

**DOI:** 10.3389/fcvm.2024.1502909

**Published:** 2024-11-29

**Authors:** Laura E. Leigh Perkins, Merry Tu

**Affiliations:** Research and Development, Abbott Vascular, Santa Clara, CA, United States

**Keywords:** percutaneous intervention, vascular closure device, hemostasis, animal models, preclinical safety

## Abstract

Vascular closure devices (VCDs) are a diverse class of cardiovascular devices intended to achieve hemostasis following arteriotomy in the common femoral artery for diagnostic and therapeutic interventional procedures. While the preclinical evaluation of VCDs parallel that of many other cardiovascular devices, there are device-specific nuances and model-specific technical considerations in assessing *in vivo* performance and handling, determining safety, and satisfying regulatory requirements. Despite the multi-decade use and continued development of novel VCD technologies, there is a paucity of published literature on their preclinical evaluation. This review intends to help mitigate this gap through a discussion of conventional animal models, their attributes and limitations, and standards in the *in vivo* assessment of performance and safety of VCDs.

## 1 Introduction

The common femoral artery (CFA) is a principal means for percutaneous access in both diagnostic and therapeutic interventional procedures. While manual compression has been the “gold standard” for achieving hemostasis following these procedures, it requires prolonged compression and bed rest, which can be time- and labor-consuming and uncomfortable for the patient. Further, high complication rates and special considerations, such as procedures necessitating large bore access sites and patients with high body habitus, on aggressive anticoagulation or antiplatelet therapy, and/or in which extended bed rest is undesirable, have elicited the need for alternatives to standard manual compression (1–3). In addition, manual compression can be considered a safe option for up to 6–8 Fr introducer size, while aortic procedures [e.g., (thoracic) endovascular aneurysm repair, transcatheter aortic valve replacement] require large bore sheath sizes and have been largely performed through open exposure of the CFA (4).

Since their first introduction in the 1990s, vascular closure devices (VCDs) are increasingly used in the clinical setting by reducing time to hemostasis, time to ambulation, and patient pain and discomfort relative to manual compression (5). However, complications still arise with their use, including but not limited to, groin hematoma, pseudoaneurysm, site thrombosis, infection, embolism, and limb ischemia (2, 6). Additionally, VCDs may not be suitable in higher risk settings of morbid obesity, small femoral arteries, femoral artery disease, or heavy arterial calcification (1). With the ambition of reducing complications and addressing unmet needs, the landscape of VCDs continues to evolve, blossoming to include a diversity of intra- and extravascular approaches to achieve rapid hemostasis for small bore (5–8 Fr) and large bore (≥12 Fr) access sites (2, 7, 8).

As with other implantable devices, preclinical evaluation is requisite to ensure product performance and safety prior to clinical use. Yet despite the decades-long history, diversity, and continued evolution of VCDs, there is a paucity of published data on their preclinical evaluation. This review is intended to mitigate this gap through a discussion of conventional animal models, their attributes and limitations, and standards for *in vivo* assessment of performance and safety of VCDs. While the focus herein is on VCDs designed for closure of arteriotomies, the concepts parallel those for VCDs intended for the closure of venotomies (9).

## 2 Overview of device-based approaches to femoral arteriotomy closure

**Figure 1:**
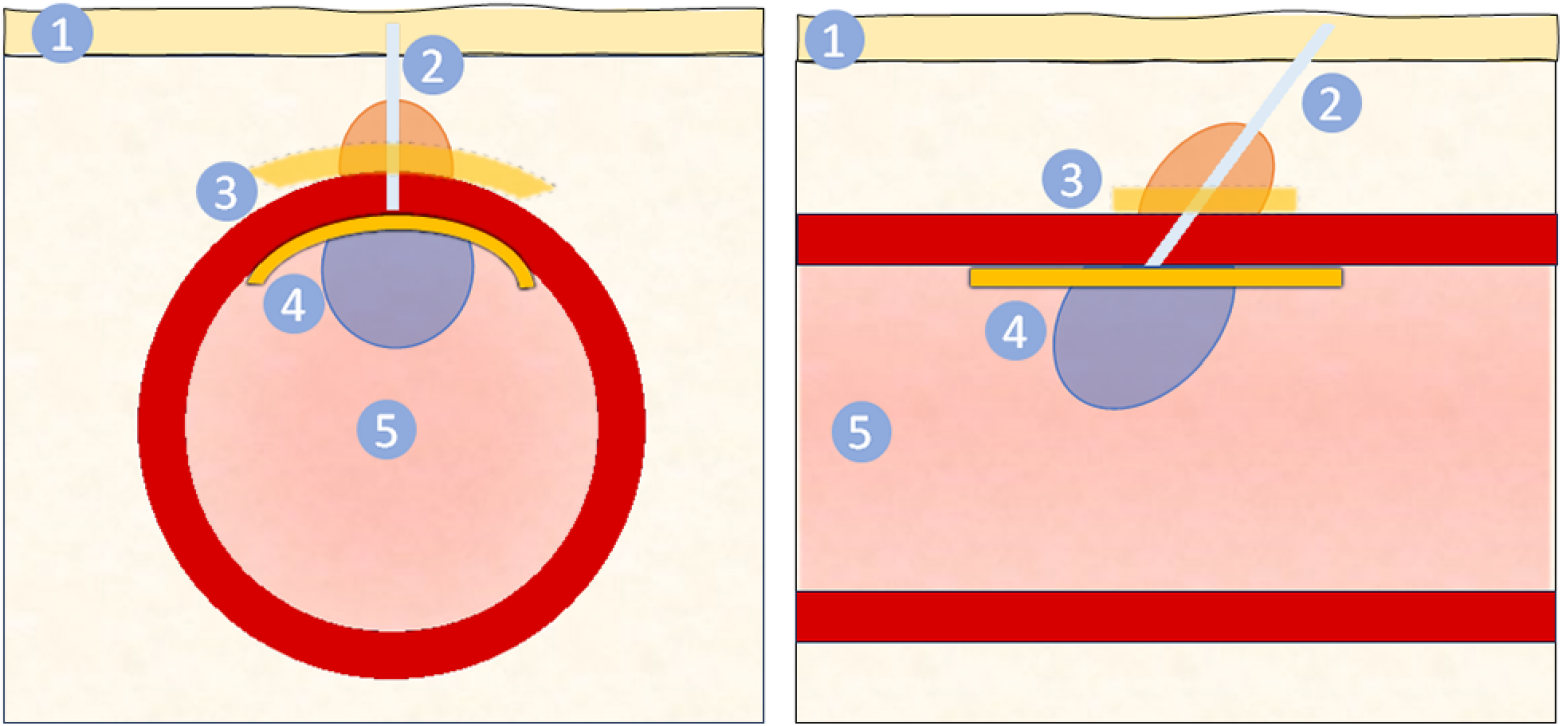
Schematic of vascular closure device from cross sectional (left) and longitudinal (right) views. The VCD is delivered through via the puncture site through the skin (1) and subcutaneous tissue, forming a tissue tract (2). This tract may contain suture or other material. For closure of the arteriotomy site, a VCD may include an extravascular component (3) and/or an endovascular component (4). The extravascular component may be a collagen plug (orange), seal, gel, or patch (yellow) that promotes hemostasis. The endovascular component (4) covers the arteriotomy site, mechanically preventing blood from exiting while ensuring patency of the artery lumen (5) and may either be a balloon (blue) or seal (yellow). The longitudinal view (right) illustrates the conventional angle of deployment from the skin to the artery, resulting in components being implanted slightly misaligned. Note the figure is representative and not to scale.

VCDs employ a spectrum of approaches to reduce the time to hemostasis. As outlined in Table 1, based on VCDs currently marketed or those in development, these approaches can be provisionally divided into (a) active vs. passive, whereby the former physically closes or covers the arteriotomy site and the latter simply facilitates natural hemostatic mechanisms; (b) foreign body vs. none, based on the presence or absence of a foreign material remaining in the body; (c) extravascular vs. endovascular; and (d) temporary vs. permanent (7). Notably, for the intents of this review, the focus is on VCDs with the presence of a foreign body, whether transient or permanent. This therefore excludes external compression devices that do not involve implant of a foreign material and are truly a device for hemostasis, not vascular closure. With the diversity of VCDs, there is no one benchmark for comparison. This is influential in the selection of a comparable control article for use in preclinical evaluation as will be discussed. A representative VCD, illustrating the gamut of “conventional” components, is provided in Figure 1. While to date there are no marketed VCDs classified as combination products through the inclusion of pharmaceuticals or biologics to influence hemostasis and/or vascular healing, aspects in the preclinical assessment of this added complexity are relevant to other combination products for which references are provided (10–12).

**Table 1:** Device-based approaches to femoral arteriotomy closure.

	Clip-based	Suture-based	Collagen-plug	“Sandwich”	Sealant, gel, or patch^a^
Active vs. passive	Active	Active	Active/Passive	Active	Passive
Foreign body	Yes	Yes	Yes	Yes	Yes
Endovascular	No	Yes	Yes	Yes	(Yes)
Extravascular	Yes	Yes	Yes	Yes	(Yes)
Temporary^b^	No	No	Yes	Yes	Yes
Permanent	Yes	Yes	No	No	No

^a^ This class may work either through placement of an endovascular (intramural) or extravascular component that facilitates natural hemostatic mechanisms but does not actively close or appose the arteriotomy site.

^b^ Including those that are transient until hemostasis is achieved (e.g., balloon) or are resorbable over time.

## 3 Animal models for the evaluation of VCDs

Akin to other implantable cardiovascular devices, large animal species are the preferred models for the assessment of VCDs relative to their anatomic and physiologic similarities to humans. Published literature on the preclinical assessment of VCDs have predominantly entailed the use of canine, porcine, and small ruminant (caprine, ovine) models, though canine is considered a less favorable species relative to high fibrinolytic activity and resistance to neointimal formation (10, 13).

The human CFA averages 6.6 mm (3.9–8.9 mm) in diameter (14), with the superficial femoral arteries of suitably-sized swine and sheep being of comparable diameter (15–17). However, as porcine have a propensity for vasospasm (13, 18, 19), sheep may be more ideal in preclinical testing, especially considering that multiple VCDs can and should be deployed within the same artery to reduce total animal usage while meeting essential endpoints. A further consideration with swine is the propensity for rapid growth of domestic strains, with mini-swine breeds often being employed in studies exceeding 90 days; study duration should therefore be considered in model and species selection especially for VCDs with components with prolonged resorption times (10, 20, 21).

**Figure 2:**
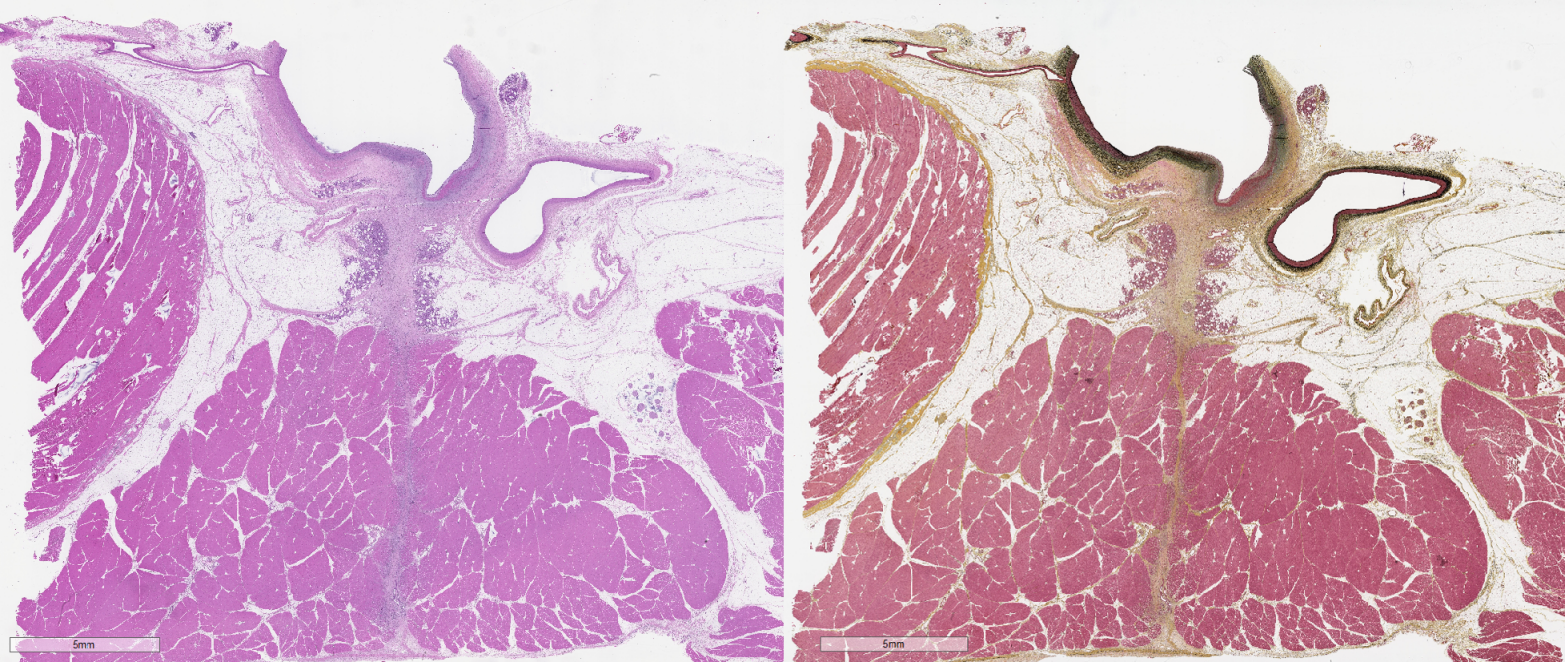
Photomicrographs of arteriotomy site in sheep femoral artery 14 days after femoral arteriotomy stained with hematoxylin and eosin (left) and Movat's Pentachrome (right). The femoral artery (FA) is at top, with the injury induced by the arteriotomy is overlain by bracket. The sartorius muscle (SM), not present in humans, overlays the FA, with a tissue tract (dashed line) coursing through the muscle. Histomorphological evaluation should include the assessment of responses in the femoral artery proper as well as in the adjacent tissue as appropriate for the SHVCD depending on the presence of endovascular and/or extravascular components.

**Figure 3:**
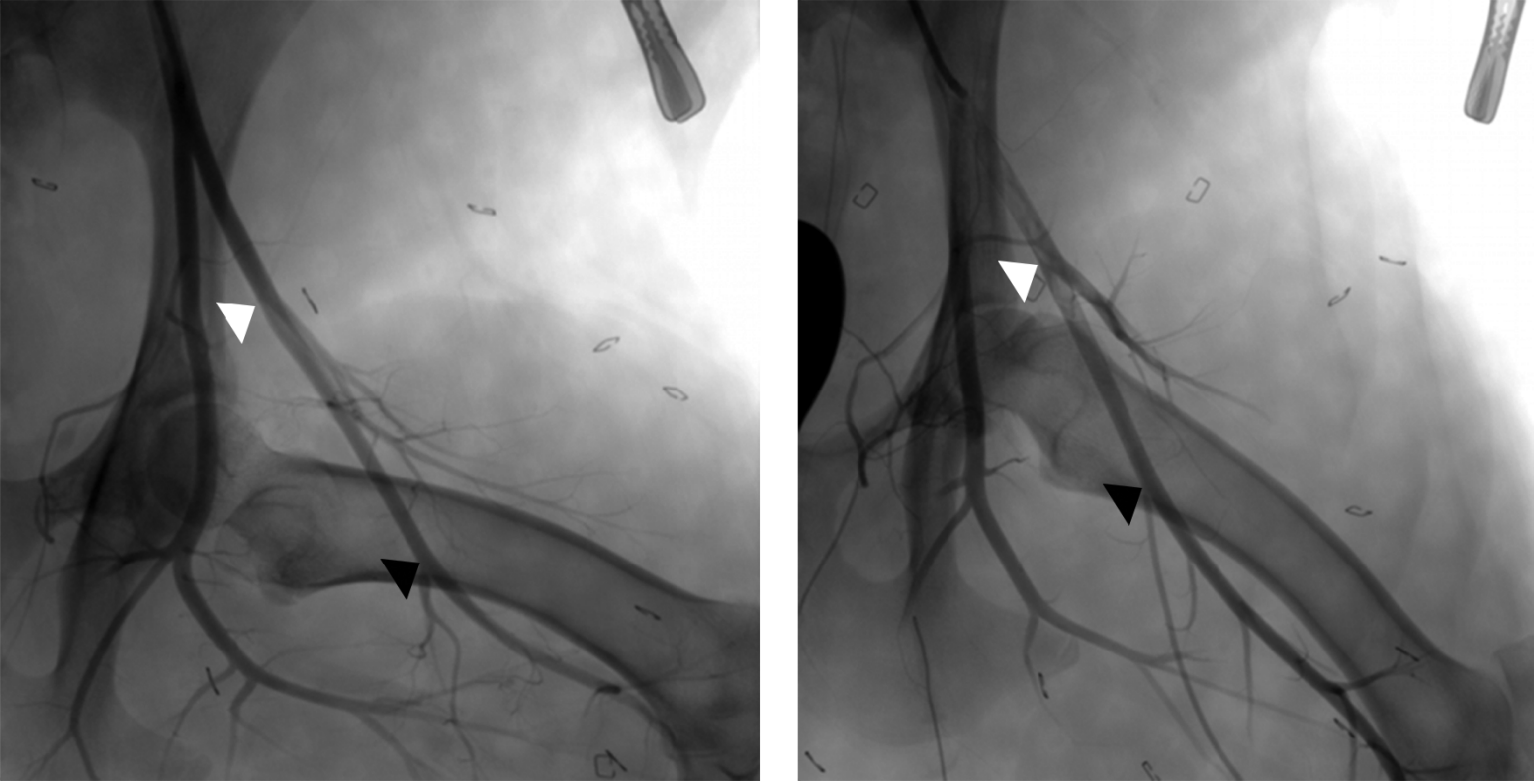
Angiographic images of normal left superficial femoral arteries with hindlimb in variable positions between partial flexion (left) and extension (right). This illustrates the variable course of the superficial femoral artery relative to the femur and the potential dimension changes (e.g. length, diameter) that can occur with alterations of leg positioning. The arrowheads denote the common region of the superficial femoral artery which is accessible for VCD implant, from the lateral circumflex artery (white) to the saphenous artery (black).

Essential to consider are aspects of clinical CFA access vs. preclinical access in the superficial femoral artery. Clinically, femoral arteriotomy is optimally performed in the CFA, which lies superficial within the femoral triangle and is contained within the femoral sheath (7). The CFA overlies the femoral head, which provides a firm base for manual compression, and the confinement of the femoral sheath can provide a secondary means of limiting access site hemorrhage. However, there are notable differences between humans and conventional quadruped models (e.g., sheep, swine). First, in humans the sartorius muscle is rudimentary and forms the lateral border of the femoral triangle. Conversely, in many quadruped species, the functional sartorius muscle is dual-headed and overlies the superficial femoral artery as it exits the femoral canal (Figure 2) (22). The presence of musculature over the femoral artery, instead of the more complaint subcutaneous adipose tissue and superficial fascia of humans, may challenge VCDs that involve deployment of an extravascular component due to the muscle's resistance to insertion. This may result in VCD compromise or failure and/or, if attempts are made to counter this resistance during implant, can place undue tension on the arteriotomy site resulting in further arterial injury or overt device failure (13). Next, in conventional quadruped models, femoral access is obtained below the inguinal crease in the superficial femoral artery, which courses distal to the femoral head. As there is less palpable support of the superficial femoral artery, attempts to achieve hemostasis via manual compression, if needed, may be more prolonged in conventional animal models as compared to the clinical setting (Figure 3) (23). Adding to this is that access in the superficial femoral artery of quadrupeds is distal to the femoral sheath, increasing the inherent risk of hematoma formation, which may be life-threatening, with subpar hemostasis imparted by the VCD being evaluated. Even with acute VCD success, animals should be monitored closely during and for the 24 h after recovery to ensure closure and hemostasis were suitably achieved.

Alternative anatomical settings have also been used in swine and small ruminants, including the infrarenal aorta of swine (24–26) and carotid arteries of sheep and goats (27–29), though surgical access is necessary for both of these anatomical locations. The large diameter of the infrarenal aorta can serve as an ideal setting for the assessment of large bore closure devices with the avoidance of vasospasm of smaller muscular arteries, and the available length can easily allow for multiple devices to be deployed (17, 30). However, there are considerations specific for the infrarenal aorta, the first of which being that the aorta is an elastic artery and may differ in response to VCD implantation as compared to the transitional muscular CFA of humans and muscular superficial femoral artery of animals (31). Second, the presence of a VCD's extravascular components within the abdominal cavity or retroperitoneal space may elicit local tissue responses disparate from those that would be obtained within the intended subcutaneous tissue with clinical use. In these situations, the use of an approved control article serves as a baseline for comparing the heightened tissue response inherent to this setting. Due to the location and required surgical manipulation to access the infrarenal aorta and carotid artery, manual compression, if needed, may not be practicable; further, device failure after closure can result in retroperitoneal or abdominal hemorrhage that may be difficult to note clinically except by mortality. This again highlights that species and location selection should be carefully considered to ensure applicable results in demonstrating performance and safety for the intended clinical use.

## 4 Technical considerations in preclinical evaluation

In addition to the complexities of suitably matching the VCD and the animal model, there are multiple aspects in the program design and study execution that are necessary to consider for implantable VCDs. Despite the undeniable diversity of VCDs, applicable across this class of devices are multiple technical aspects that guide success in the *in vivo* assessment of a VCD. These multiple, often interdependent, considerations are outlined in Table 2 and are discussed in the following.

**Table 2:** Technical considerations in the preclinical evaluation of vascular closure devices (VCDs).

Anatomy	Implantation	Study/Program design
Model selection (species, anatomical location)	Activated clotting time (ACT)	VCD mechanism of action.
Vessel dimensions (diameter, length)	Operator experience in arteriotomy (femoral or other)	Study objectives: feasibility, characterization (e.g., degradation), performance, safety, biocompatibility.
Leg positioning	Operator experience with VCD	Time points.
VCD dimensions (arteriotomy site)	Ultrasound guidance	Number of implants per time point.
Inter-device spacing (for multiple implants)	Use of vascular spasmolytics	Number of animals per time point.
		Control device selection.

As an overarching concept in the preclinical evaluation of all medical devices, efforts should be made to follow, as closely as reasonably possible, use conditions as outlined in the product's instructions for use (IFU). This compliance is notably relevant for studies intended for regulatory submission, for which justifications should be provided for deviations from the IFU. Further, a risk analysis is recommended to ensure that risks specific to the VCD be accordingly evaluated in the preclinical setting (32).

### 4.1 Anatomy

Pertinent anatomical considerations comparing bipeds with standard quadruped models have been discussed; additional considerations include vessel diameter, dimensions of the VCD and arteriotomy site, and leg positioning. To access the femoral arteries, animals are placed in supine position; as such, consistent leg positioning and securement in moderate abduction helps ensure accuracy and consistency in angiographic measurement of vessel diameters not only during implant procedures but also at follow-up when percent diameter stenosis is included as an angiographic endpoint (Figure 3) (33). The length of the superficial femoral artery accessible for arteriotomy commonly allows for multiple VCDs to be used in a single femoral artery. In such cases, the arterial length available needs to accommodate the length of the implant(s) and allow for adequate spacing between implants. This inter-device spacing should account for both arterial deformation induced by natural movement (34) and device angulation during deployment, which in the clinical setting commonly approximates a 45°–60° angle or 2 cm between the skin and the intended arteriotomy site.

### 4.2 Implantation

Prior to and during implantation of cardiovascular devices, the activated clotting time (ACT) should be monitored to ensure values align to that of the product's IFU. As VCDs are used to provide hemostasis of the arteriotomy following completion of an interventional procedure, the targeted ACT range is typically lower (e.g., 150–300 s) relative to other cardiovascular devices (e.g., 250–400 s).

Achieving successful hemostasis while minimizing complications relies on operator experience both in arteriotomy in the selected model and in the use of the VCD being evaluated. Analogous to other medical devices, there may be a steep learning curve in proper use which is, as with other cardiovascular devices, “intricately linked to study outcomes” (32, 35). Operators should therefore be properly trained prior to the execution of a preclinical study, which may require feasibility studies prior to execution of studies intended for regulatory submission. VCDs align with other implantable medical devices in the criticality of the selection of proper device size and technique for access and deployment, as well as for manual compression as needed without VCD compromise (36, 37). Dual operators may be required in preclinical studies to ensure suitable expertise both in *in vivo* procedures and in assessing device performance and handling as it relates to clinical use.

Clinically the use of ultrasound guidance is recommended with femoral access (38), and the same verification is beneficial in preclinical studies to document accurate deployment within the superficial femoral artery. As femoral arteriotomy involves full thickness injury, a VCD may further exacerbate acute arterial irritation and injury, resulting in focal to segmental vasospasm. Vascular spasmolytics, such as vasodilators and/or calcium channel blockers, should be included as preventative and/or therapeutic strategies. Following deployment, successful closure of the arteriotomy site and vessel patency should be confirmed.

### 4.3 Study/program design

In the design of a preclinical study and the overarching program for evaluation of a VCD, an understanding of the VCD components and mechanism of action for achieving hemostasis is imperative. As discussed in the following, this understanding influences the types of studies required, study durations, and control article selection. Recognizing the 3R's animal welfare framework (39), efforts to minimize the number of animals while ensuring robust and reproducible findings applicable across multiple requisite endpoints should be employed. With proper attention to study design, studies in compliance with Good Laboratory Practices in large animal models can fulfill endpoints required for ISO10993 parts 4, 6, and 11, effectively minimizing total animal usage (40–42).

All preclinical studies are to start with well-defined objectives. Feasibility (proof of concept), characterization of the degradation profile, and/or safety (biocompatibility) serve as standard objectives; performance and handling, including efficacy at hemostasis, are commonly employed as additional secondary objectives in each study (32, 42). While conducted in a biological setting that is not always predictable, study objectives should be supported by well-defined endpoints or acceptance criteria that concretely define device success or failure.

Safety studies may entail acute (≤3 days), 4 weeks, and 12 weeks durations to align to critical phases of healing and to satisfy safety and biocompatibility requirements; however, this is dependent on the VCD materials, mechanism of action, and duration to resorption. Additional and/or longer time points are generally required to demonstrate tissue compatibility throughout the degradation process until near to complete resorption and tissue quiescence are achieved. For resorbable VCDs, time points for degradation and safety studies are ideally aligned to be able to specify the local tissue response at critical times in the degradation process. In early phase feasibility studies, staggering the time of implants can allow for one animal to suffice for two follow-up time points.

With the diversity in design and mechanism of action across VCDs, control articles for use in preclinical testing should be carefully selected based on their congruence with the test article. Due to the diversity of and new developments in VCDs, a suitable control article may not be available, and exclusion of a control article may be justified. Randomization, the number of implants, and the number of animals used should be based on scientifically sound rationale that will adequately fulfill the desired endpoints (32, 43).

## 5 Modalities and methods for assessing performance and safety

The *in vivo* assessment of VCD parallels that of other implantable cardiovascular devices, reviews for which are provided (37, 44, 45). While performance and handling primarily address the acute phase of deployment with the “VCD system” [delivery system and implantable component(s)], safety holistically covers implant through chronic endpoints as dictated by the implantable VCD components.

### 5.1 Performance considerations

Often serving as a secondary endpoint to safety, VCD performance and handling can be readily assessed in preclinical studies while bearing in mind the limitations detailed previously. This includes specifics aligned, but not limited to, the design of the VCD and its deliverability, deployment, and removal (e.g., compatibility with other devices, ease of access and deployment, ease of delivery system removal); acute angiographic assessment of the vascular response (e.g., vasospasm, vessel dissection, thrombosis and/or embolization); and time to hemostasis. Per FDA recommendations, these assessments should ideally be performed by practitioners with clinical experience, though as alluded to previously, the learning curve with a VCD may be preclusive thus allowing personnel experienced with the VCD being evaluated to perform this evaluation (32). While time to hemostasis is the driver of VCDs, there may be model specific, complicating factors that limit the success of a VCD despite its likely clinical performance and efficacy. Whether this includes difficulty in successfully deploying components properly through the overlying sartorius muscle to adequately secure the VCD as clinically intended or includes excess tension being applied to the superficial femoral artery that induces additional injury, severe vasospasm, and/or localized thrombosis, these observations are to be recorded but may be justified based on the nature of the VCD and model. And while this performance endpoint focuses primarily on acute outcomes, the subversive effects of inadequate VCD apposition with leg movement with arterial deformity in a standard quadruped stance must be considered in the totality of device performance and justified. Herein *in vivo* imaging, as discussed in the following, may be an asset as part of the acute performance assessment.

Also applicable under device performance assessment is *in vivo* thrombogenicity testing in accordance with ISO 10993-Part 4 (46). This includes both the limited contact delivery system as well as any components of the VCD that are in direct contact with blood, the latter of which is addressed under evaluation of local effects. Including this endpoint in safety studies is more closely aligned with clinical use and avoids some of the challenges associated with prescribed arterial or venous implant protocols in canines.

### 5.2 *In vivo* imaging

Inclusive of device performance and handling, angiographic imaging in preclinical studies gives early and clinically relevant insight into how a VCD may be expected to perform clinically. Angiography is used respectively pre- and post-deployment to identify the target implant location and to assess vessel patency, contrast leakage (perforation), dissection, vasospasm, thrombosis and thromboembolization. This modality is limited by its two-dimensional nature for which intravascular imaging, including intravascular ultrasound (IVUS) and optical coherence tomography (OCT), can serve as useful supplements, especially for VCDs with endovascular component(s) that last beyond the acute time frame. These intravascular imaging modalities allow for discernment of features including vessel patency, VCD apposition, and VCD characteristics and have been shown to provide valuable information when used for interim assessments while deferring the need for animal termination for histological evaluation (47). While OCT has higher resolution than IVUS, it is limited in the depth of penetration and may not be suitable in superficial femoral arteries greater than −5 mm diameter unless the imaging catheter is properly aligned over the VCD intravascular component (48).

### 5.3 Evaluation for systemic safety

Monitoring of the health and well-being of test systems over the course of a preclinical study is standard humane practice for animals used in biomedical research. This monitoring also is essential as any relevant study observations must be able to be discerned as spontaneous, related to the procedure, and/or related to the VCD. Systemic safety is assessed through daily and periodic monitoring of clinical signs, through clinical pathology (pre-implant and at termination), and through the gross and histological evaluation of organs distal from the implant site (32, 49). For VCDs implanted in femoral and aortic locations, this importantly includes hindlimb skeletal muscles, skin, and the coronary band. As true for all medical devices, regional draining lymph nodes also should be collected for histological evaluation (50).

### 5.4 Evaluation of local effects

The evaluation of local effects spans from gross assessment (necropsy) to the histological evaluation of stained tissue sections. Proficiency in the former substantially benefits the latter, with interim steps that also influence histological evaluation and the ultimate interpretation regarding the safety and effectiveness of a VCD. Critically, as true for other medical devices, maintaining the integrity of the device-tissue interface throughout this multistep process is essential.

Publications highlighting important considerations in necropsy, sample procurement, fixation, and processing for histological evaluation of both permanent and bioresorbable medical devices, and thus largely applicable to VCDs, are provided for reference (51–53). And though the diversity of VCDs (Table 1) precludes a singular approach applicable across this class of cardiovascular devices, there are notable commonalities. First, prior to dissection and isolation of the region of interest, vessels should be thoroughly flushed with a physiological solution (e.g., saline, LRS) to remove blood from the artery. Arteries intended for histological evaluation should then be perfusion fixed for a sufficient duration, most commonly with neutral buffered formalin; samples intended for degradation analysis should only be flushed without exposure to fixative. The combined use of skin tattoos, angiographic imaging, and grossly visible markers aids in defining the territory of implantation and subsequently locating implanted VCD components, especially for resorbable materials late in the resorption process. The procured tissue of interest should include *en toto* adequate naïve reference vessel (proximal and distal to implant sites), the implanted region, and, for VCDs with extravascular components, tissues overlying the artery which can later undergo detailed dissection or tissue sectioning. Of note, VCDs are deployed in alignment with the introducer sheath used during the access procedure, whereby skin insertion to the arteriotomy site commonly estimates a 45°–60° angle or targets −2 cm from the skin surface to the arterial insertion (Figure 1). As the skin entry site does not align to the arteriotomy site, considerations of this natural angulation are to be accounted for in tissue procurement and in sectioning for histological evaluation to ensure all VCD components (endo- and extravascular) are included. Further, VCDs are generally of modest size as they are intended to close access sites typically from −1.5–8 mm (4–24 Fr), and the discernibility of components is time dependent with resorbable VCDs. Thus, an understanding of the VCD design and mechanism of action is imperative to ensuring proper methods are employed in tissue collection, trimming, and sectioning.

Histological evaluation involves both semi-quantitative and qualitative assessments; semi-quantitative assessment allows for numerical and/or statistical enumeration (i.e., test vs. control article) of pre-specified parameters, and the qualitative assessment elucidates characteristics and nuances that integrally play into the ultimate interpretation of safety. Importantly, statistical differences between study articles do not necessarily imply biological significance, and this should be reflected in reporting. Quantitative morphometry data may be obtained but should be interpreted with caution due to tissue changes incurred post-mortem and during tissue processing; angiographic imaging (or other *in vivo* imaging modality) is more clinically relevant and accurate. Standardized schematics in histological evaluation have been published for other cardiovascular devices (10, 11), and while the diversity of VCDs poses challenges to a singular standardized approach, there are unified parameters to consider as outlined in Table 3. While it is the totality of the tissue response being assessed, histological parameters are subjectively divided to assess responses of the artery and responses within the overlying tissue relative to the arteriotomy and to the endovascular and/or extravascular VCD components, as applicable (Figure 2). Again, and importantly, all steps of the process—from the tissue sampling and handling, processing, trimming, and sectioning to histological evaluation - combine to allow for proper interpretation as to the safety of the VCD within the animal model utilized.

**Table 3:** Suggested histological parameters for the assessment of the local tissue response to vascular closure devices (VCDs).

Quantitative	Comments/Qualitative aspects
Artery/Endovascular components	
Thrombus (luminal, mural)	Composition and duration, occlusive or non-occlusive.
Endothelialization/Tissue coverage	Endothelial denudation is not uncommon with handling and should be interpreted in this context.
Neointimal formation (intraarterial fibrosis/granulation tissue)	Amount, composition, and maturity relative to duration post-implantation. Note that contracture of the tissue often exacerbates neointimal thickness and luminal occlusion.
Arterial injury	Alignment of VCD components to arteriotomy site.
	Degree of injury appropriate to arteriotomy, or pronounced due to tissue response to VCD (e.g., medial SMC loss, leukocytic infiltrates).
Medial SMC loss (myonecrosis), mineralization	Discerned whether from compression injury, segmental vasospasm, and/or cytotoxicity of VCD components.
Leukocytic infiltrates (inflammation)	Cell types and relative composition provide insight into duration and whether active or resolving.
Multinucleated giant cells	Specific for foreign body response; morphology and location relative to VCD components.
Medial fibrosis	As an appropriate response to arteriotomy or the result of extensive medial myonecrosis.
Neovascularization	Resultant from localized tissue hypoxia.
Tissue tract/Extravascular components	
Hemorrhage/Hemosiderophages	Natural aspect in the acute phase following arteriotomy, the presence of extravasated erythrocytes should be transient without extensive accumulation of hemosiderophages.
Skeletal myocyte loss (myonecrosis), Mineralization	Discerned whether from arteriotomy or other injury, or whether related to cytotoxicity of VCD components.
Adipose saponification (fat necrosis)	Discerned whether from arteriotomy or other injury, or whether related to cytotoxicity of VCD components.
Leukocytic infiltrates (inflammation)	Cell types and relative composition provide insight into duration and whether active or resolving.
Fibrosis	Adventitial, as it relates to arteriotomy injury, as well as around extravascular VCD components, if applicable.
Neovascularization	Resultant from localized tissue hypoxia.

### 5.5 Assessment of resorption

As patients may require multiple accesses to be performed in the CFA, the use of transient, bioresorbable materials in VCDs that can allow for later re-access is a desirable feature. Advances in synthesis and characterization techniques have resulted in a diversity of polymers in which mechanical, physiochemical, and degradation properties can be tailored to VCD applications (54). The characterization of the *in vivo* degradation behavior of bioresorbable components is complimentary to histological evaluation, providing an understanding of the tissue response throughout the course of resorption, including critically during peak mass loss when there is heightened potential for leukocytic involvement (inflammation). Histological evaluation also is adjunctive to analytical results by illustrating characteristic changes in the material that occur *in vivo*, such as erosion, tinctorial changes with histological stains, and discontinuities or fragmentation, the latter of which can be influenced by stresses and strains on the material and/or as a natural feature of the resorption process. Concordant time points should thus be used for safety studies and studies for degradation characterization. As histological assessment is based on a histochemically-stained thin slices (4–5 µm) obtained from a three-dimensional object, observed histomorphologic changes should be described based on appearance and not overinterpreted as evidence of resorption, which can only truly be determined by chemical analysis.

## 6 Limitations

Preclinical models allow for thorough assessment in an *in vivo* setting to provide a reasonable assurance of performance and safety of a VCD before clinical use. Regulatory studies are generally conducted in normal animals with naïve arteries, ensuring uniformity that facilitates interpretation of study results. Thus, there are limitations considering the complexities faced in clinical use such as heavy arterial calcification, femoral artery disease, and extremes of body habitus (high or low BMI). To address these acute anatomical concerns, perfused cadaveric models can fill the void to assess feasibility for use in the context of these human specific states which are difficult to emulate in preclinical models (55). A means of standardized assessment in cadaveric models can thus serve as a valuable tool to reduce animal use and enhance the development of VCDs to address these difficult contexts which are becoming more common place in the cardiovascular arena.

Another critical limitation of conventional preclinical models is the assessment patient comfort at the implant site, spanning from acute to chronic. This crucial component extends beyond safety and cannot be addressed by animal models aside from a cursory assessment for lameness (56). Across the vast range of cardiovascular devices, considering their broad usage, the assessment of patient comfort is most notable to VCDs, again inclusive of not only the acute time during deployment and ambulation, but also throughout the time that the VCD is considered a relevant implant. Thus, preclinical studies give a reasonable assurance of performance and safety for VCDs, but only the clinical setting, with proper data collection, can provide results as to how a VCD performs in achieving hemostasis, its adequacy in subset populations, and its ability to reduce patient discomfort beyond extended manual compression and bed-rest requisite of manual compression.

## 7 Discussion

While spanning greater than four decades of use, VCDs continue to evolve to address the expanding volume, needs, and patient complexities entailed with interventional procedures conducted via common femoral arteriotomy. Preclinical models are optimized to assess and give a reasonable assurance of performance and safety prior to clinical use, though there are considerations between the use of quadrupeds in devices intended for bipeds, and in the broadening of results from normotensive, average weight animals with normal hemostasis and naïve, uncalcified arteries to that of humans in which conditions often deviate from normality. Still, inappropriate use of these “accessory” devices can have dire consequences without proper assessment of risk, inclusive of performance and safety, in the preclinical setting. Accurate reporting on the results of preclinical studies, and especially those intended for regulatory submissions, requires a synthesis of information obtained across evaluation modalities and disciplines. As true for other cardiovascular devices, the evaluation of VCDs is a collaborative effort, requiring multidisciplinary input from the engineers, operators, pathologists, and personnel with expertise in animal health and husbandry to ultimately determine *in vivo* performance and safety of a VCD prior to clinical use.
